# Patterns and prevalence of dyslipidemia in patients with different etiologies of chronic liver disease

**DOI:** 10.1007/s00508-019-01544-5

**Published:** 2019-09-06

**Authors:** Lukas W. Unger, Bernadette Forstner, Stephan Schneglberger, Moritz Muckenhuber, Ernst Eigenbauer, Bernhard Scheiner, Mattias Mandorfer, Michael Trauner, Thomas Reiberger

**Affiliations:** 1 Division of General Surgery, Department of Surgery, Medical University of Vienna, Währinger Gürtel 18–20, Vienna, Austria; 2 IT-Systems & Communications, Medical University of Vienna, Währinger Gürtel 18–20, Vienna, Austria; 3 Division of Gastroenterology and Hepatology, Department of Internal Medicine III, Medical University of Vienna, Währinger Gürtel 18–20, 1090 Vienna, Austria; 4 Vienna Hepatic Hemodynamic Laboratory, Medical University of Vienna, Währinger Gürtel 18–20, Vienna, Austria

**Keywords:** Portal hypertension, Serum lipid levels, Hypercholesterolemia, Hypertriglyceridemia, Cirrhosis

## Abstract

**Background:**

Liver disease impacts on hepatic synthesis of lipoproteins and lipogenesis but data on dyslipidemia during disease progression are limited. We assessed the patterns of dyslipidemia in (i) different liver disease etiologies and discriminated (ii) non-advanced (non-ACLD) from advanced chronic liver disease (ACLD) as it is unclear how progression to ACLD impacts on dyslipidemia-associated cardiovascular risk.

**Methods:**

Patients with alcoholic liver disease (*n* = 121), hepatitis C (*n* = 1438), hepatitis B (*n* = 384), metabolic/fatty liver disease (*n* = 532), cholestatic liver disease (*n* = 119), and autoimmune hepatitis (*n* = 114) were included. Liver stiffness ≥15 kPa defined ACLD. Dyslipidemia was defined as total cholesterol >200 mg/dL, low-density lipoprotein (LDL)-cholesterol >130 mg/dL and triglycerides >200 mg/dL.

**Results:**

Across all etiologies, total cholesterol levels were significantly lower in ACLD, when compared to non-ACLD. Accordingly, LDL-cholesterol levels were significantly lower in ACLD due to hepatitis C, hepatitis B, metabolic/fatty liver disease and autoimmune hepatitis. Triglyceride levels did not differ due to disease severity in any etiology. Despite lower total and LDL cholesterol levels in ACLD, etiology-specific dyslipidemia patterns remained similar to non-ACLD. Contrary to this “improved” lipid status in ACLD, cardiovascular comorbidities were more prevalent in ACLD: arterial hypertension was present in 26.6% of non-ACLD and in 55.4% of ACLD patients (*p* < 0.001), and diabetes was present in 8.1% of non-ACLD and 25.6% of ACLD patients (*p* < 0.001).

**Conclusion:**

Liver disease etiology is a major determinant of dyslipidemia patterns and prevalence. Progression to ACLD “improves” serum lipid levels while arterial hypertension and diabetes mellitus are more prevalent. Future studies should evaluate cardiovascular events after ACLD-induced “improvement” of dyslipidemia.

**Electronic supplementary material:**

The online version of this article (10.1007/s00508-019-01544-5) contains supplementary material, which is available to authorized users.

## Introduction

Dyslipidemia, among other factors, is a major risk factor for cardiovascular disease (CVD) development and progression [1] and therefore, guidelines recommend lipid-lowering therapy in patients at increased risk for CVD. As the liver plays a central role in lipid metabolism [2], lipid profiles are altered by chronic liver disease (CLD) severity. Although a vast body of literature exists on the effect of CLD on lipid profiles in several etiologies, state of the art assessment of CLD severity by, e.g. grading fibrosis severity is lacking in many studies and advanced CLD (ACLD)/cirrhosis is usually classified as a distinct etiology [2]. This classification is insufficient, as dyslipidemia patterns change during ACLD, and baseline values are strongly dependent on the underlying etiology. In addition, concomitant CVD risk factors such as arterial hypertension and diabetes mellitus must be taken into account when assessing the overall CVD risk.

Nowadays, non-invasive methods such as transient elastography are available to reliably assess the severity of fibrosis and guide/monitor CLD severity [3]. Therefore, evaluation of fibrosis severity in large patient cohorts is feasible and allows correlation with readily available lipid profiles. Actual real-life data are therefore warranted as treatment in chronic liver disease has evolved and most patients are exposed to an altered lipid profile for an extensive period of time as progression to ACLD can be halted in many cases. In addition, although cut-off levels for initiation of lipid-lowering treatment/statin treatment are well-established and stratified by clinical atherosclerotic cardiovascular disease risk, no specific guidelines for CLD and concomitant CVD are available.

Despite altered metabolism of statins, the most common drug class used as lipid-lowering therapy, and an increased risk of rhabdomyolysis [4, 5], hepatotoxic effects and severe adverse events are lower than suggested in the past [4]. Nevertheless, statins remain underutilized in patients with non-ACLD [6]. This gap in clinical implementation of guidelines might further impair outcome in CLD as several studies have shown beneficial effects on portal hypertension of statin therapy in CLD irrespective of low-density lipoprotein (LDL)-cholesterol levels [7]. Until further evidence from prospective randomized controlled trials is available, real-life data in dyslipidemia patterns and severity with respect to CLD etiology and severity is needed. Therefore, this study aimed to investigate differences among the most common CLD etiologies, namely alcoholic liver disease (ALD), non-alcoholic fatty liver disease (NAFLD), hepatitis C (HCV), hepatitis B (HBV), cholestatic liver diseases, primary sclerosing cholangitis (PSC) and primary biliary cholangitis (PBC) as well as autoimmune hepatitis (AIH).

## Patients, material and methods

### Study design and patient selection

**Fig. 1:**
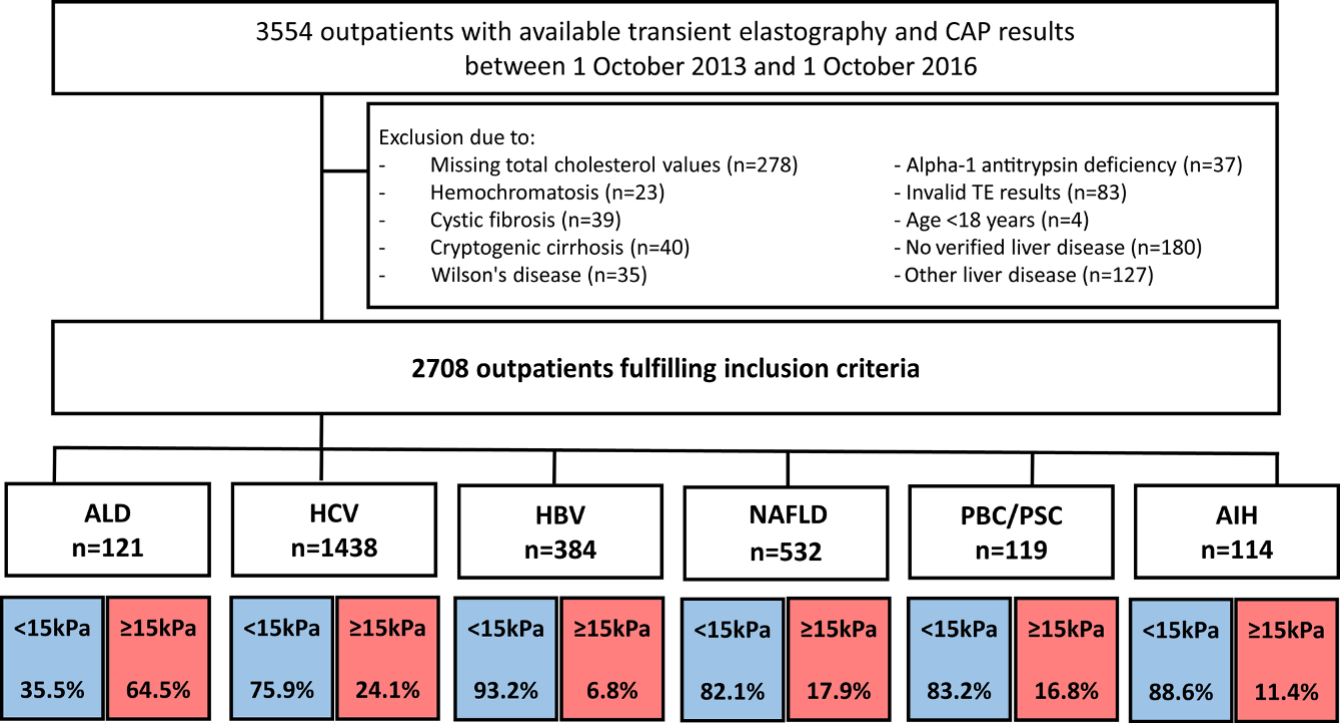
Patient consort flow chart. *AIH* autoimmune hepatitis, *ALD* alcoholic liver disease, *CAP* controlled attenuation parameters, *HBV* hepatitis B virus infection, *HCV* hepatitis C virus infection, *NAFLD* non-alcoholic fatty liver disease, *PBC* primary biliary cholangitis, *PSC* primary sclerosing cholangitis

All patients with (i) a clinically established diagnosis of ALD, HCV, HBV, NAFLD, cholestatic liver disease or AIH, (ii) available and valid liver stiffness measurements (LSM) by transient elastography (TE) with controlled attenuation parameter (CAP) values, (iii) available information on total serum cholesterol laboratory values within 3 months of TE-based liver stiffness measurement, and (iv) age >18 years presenting in the liver outpatient clinic of the Medical University of Vienna between October 2013 and October 2016 were included. After exclusion of patients that did not fulfil the inclusion criteria, 2708 patients were identified for further analysis. A patient flow chart and distribution according to liver disease severity is presented in Fig. 1.

### Assessment of liver fibrosis and hepatic steatosis

The assessment of liver stiffness using TE was carried out by experienced operators using the FibroScan® (EchoSens, Paris, France) device, as previously described [8]. Overnight fasting was a prerequisite for TE measurements and a total number of 10 valid measurements were required [9]. A cut-off value of ≥15 kPa defined ACLD, as suggested by the Baveno VI faculty consensus [10].

#### Definition of dyslipidemia

Total cholesterol levels of >200 mg/dL, LDL cholesterol levels >130 mg/dL or triglyceride levels >200 mg/dL were considered as dyslipidemia, according to the American Association of Clinical Endocrinologists and American College of Endocrinology 2017 guidelines [11]. Low high-density lipoprotein (HDL) cholesterol was not considered as dyslipidemia since there exists no treatment specifically indicated for low HDL cholesterol levels. In cases of intake of any lipid-lowering drug, the patients were excluded from the analyses of plasma lipid profiles, since lipid lowering therapy would have been a significant confounder. For the epidemiological analysis on dyslipidemia prevalence, patients on lipid-lowering therapy were considered as suffering from dyslipidemia, except patients with cholestatic liver disease and fibrates as second line therapy.

#### Evaluation of concomitant metabolic disorders

To evaluate concomitant CVD and, therefore, additional CVD risk factors, arterial hypertension as well as antihypertensive treatment and prescribed drug classes were assessed. Notably, non-selective beta blocker treatment that was solely administered for preventing portal hypertensive bleeding was not considered as antihypertensive medication.

#### Statistical analysis

Differences in proportions between groups were evaluated using χ^2^-test or Fisher’s exact test whenever appropriate. For normally distributed numerical variables and comparisons between two groups, Student’s t‑test or Mann-Whitney U-test was used, as applicable. For visualization of liver stiffness, total cholesterol levels, as well as for linear regression and D’Agostino’s K^2^-test, GraphPad Prism Version 8.1.2 (GraphPad Software, La Jolla, CA, USA) was used. SPSS Version 24 (IBM, Armonk, NY, USA) was used for all other statistical analyses. A *p*-value <0.05 denoted statistical significance.

#### Institutional review board

The retrospective cohort study was conducted according to the Declaration of Helsinki and was approved by the Medical University of Vienna’s institutional review board (EK-Nr. 2013/2016; https://ekmeduniwien.at/core/catalog/2016/).

## Results

### Differences in patient characteristics according to underlying liver disease in patients with non-ACLD and ACLD

In order to investigate differences in chronic liver disease severity, Fig. 1 presents proportions of patients with non-ACLD and ACLD. The vast majority of patients were non-ACLD patients with <15 kPa in TE except for ALD where the majority of patients suffered from ACLD. In order to assess differences between various etiologies of CLD, the characteristics of patients with non-ACLD (Table 1) as well as ACLD were compared (Table 2). Baseline characteristics were significantly different between etiologies, with the most profoundly increased concomitant CVD risk factors, namely body mass index (BMI), diabetes mellitus and arterial hypertension, in NAFLD. This pattern was preserved in ACLD, although arterial hypertension was even more prevalent in cholestatic liver disease and markedly elevated in patients suffering from HBV.

**Table 1:** Patient characteristics in non-ACLD patients (liver stiffness <15 kPa)

	ALD	HCV	HBV	NAFLD	Cholestatic	AIH
Patients (*n*)	43	1092	358	437	99	101
Age (years)	58.1 (16.3)	51.9 (19.4)	40.9 (20.7)	51 (19.8)	54.4 (18.8)	52 (25.8)
CAP (dB/m)	258 (80)	230 (75)	229 (76)	299 (72.5)	220 (64)	228.5 (73.5)
BMI (kg/m^2^)	25.1 (6.2)	24.7 (5.6)	24.9 (5.7)	28.3 (6.2)	24.4 (5.2)	25.1 (4.2)
ALP (U/l)	89.0 (73.0)	69.0 (28.3)	63.0 (26.5)	73.0 (37.5)	119.0 (70.0)	68.0 (52.6)
GGT (U/l)	140 (273)	37 (58)	22 (21)	53 (85)	91 (148)	46 (77)
AST (U/l)	37 (32)	34 (26)	28 (14)	31 (16)	31.5 (19.3)	30 (17.3)
ALT (U/l)	28 (34)	39 (43)	32 (28)	43 (36.5)	34 (32.8)	29 (32.5)
Bilirubin (mg/dl)	0.56 (0.54)	0.50 (0.32)	0.55 (0.41)	0.50 (0.35)	0.56 (0.38)	0.55 (0.31)
Albumin (g/l)	43.0 (3.2)	44.5 (4.3)	45.1 (3.8)	45.2 (4.1)	45.1 (4.5)	44.2 (3.8)
Platelet count (G/l)	199 (78)	215 (80)	213 (72)	240 (81)	252 (85)	223 (77)
INR	1.1 (0.3)	1.0 (0.1)	1.1 (0.1)	1.0 (0.2)	1.0 (0.2)	1.0 (0.1)
Arterial hypertension (%)	55.8	23.6	15.6	37.8	37.4	26.7
Diabetes mellitus (%)	11.7	5.6	3.4	15.6	9.1	8.9
Male sex (%)	83.7	61.4	60.3	50.3	29.3	24.8

Data are presented as median (IQR) for numerical variables

*AIH* autoimmune hepatitis, *ALD* alcoholic liver disease, *ALP* alkaline phosphatase, *AST* aspartate aminotransferase, *ALT* alanine aminotransferase, *BMI* body mass index, *CAP* controlled attenuation parameters, *GGT* Gamma-glutamyl transferase, *HBV* hepatitis B virus infection, *HCV* hepatitis C virus infection, *IQR* interquartil range, *NAFLD* non-alcoholic fatty liver disease

**Table 2:** Patient characteristics in ACLD patients (liver stiffness ≥15 kPa)

	ALD	HCV	HBV	NAFLD	Cholestatic	AIH
Patients (*n*)	78	346	26	95	20	13
Age (years)	56.7 (14.6)	55.7 (13.8)	52.3 (14.1)	56.4 (18.1)	59.3 (17.7)	52.5 (22.3)
CAP (dB/m)	247 (84)	249 (83)	270 (102)	321 (58)	215 (88)	266 (80)
BMI (kg/m^2^)	24.7 (5.9)	26.2 (5.4)	28.1 (8.2)	29.7 (5.8)	24.8 (6.6)	26.0 (14.9)
ALP (U/l)	100.5 (63.3)	85.5 (44.0)	81.0 (27.0)	80.0 (44.0)	166.5 (64.7)	115.5 (90.8)
GGT (U/l)	166 (229)	73 (106)	62 (58)	89 (102)	119 (192)	153 (251)
AST (U/l)	42 (30)	49 (50)	44 (26)	44 (32)	54 (66)	50 (37)
ALT (U/l)	29 (19)	47 (57)	47 (38)	45 (40)	56 (53)	33 (32)
Bilirubin (mg/dl)	1.26 (1.26)	0.77 (0.59)	0.71 (0.67)	0.68 (0.62)	1.11 (1.10)	0.73 (0.93)
Albumin (g/l)	37.5 (7.5)	41.3 (6.5)	40.8 (5.0)	43.7 (5.9)	40.0 (8.9)	42.3 (10.1)
Platelet count (G/l)	137 (122)	117 (85)	141 (130)	173 (120)	128 (97)	187 (136)
INR	1.3 (0.3)	1.2 (0.2)	1.3 (0.3)	1.1 (0.2)	1.2 (0.3)	1.0 (0.2)
Arterial hypertension (%)	64.1	52.3	57.7	57.9	65.0	46.2
Diabetes mellitus (%)	20.5	24.3	30.8	36.9	15.0	15.4
Male sex (%)	69.2	69.9	69.2	67.4	30	46.2

Data are presented as median (IQR) for numerical variables

*AIH* autoimmune hepatitis, *ALD* alcoholic liver disease, *ALP* alkaline phosphatase, *AST* aspartate aminotransferase, *ALT* alanine aminotransferase, *BMI* body mass index, *CAP* controlled attenuation parameters, *GGT* Gamma-glutamyl transferase, *HBV* hepatitis B virus infection, *HCV* hepatitis C virus infection, *IQR* interquartil range, *NAFLD* non-alcoholic fatty liver disease

### Differences in serum lipid levels in non-ACLD and ACLD

**Fig. 2:**
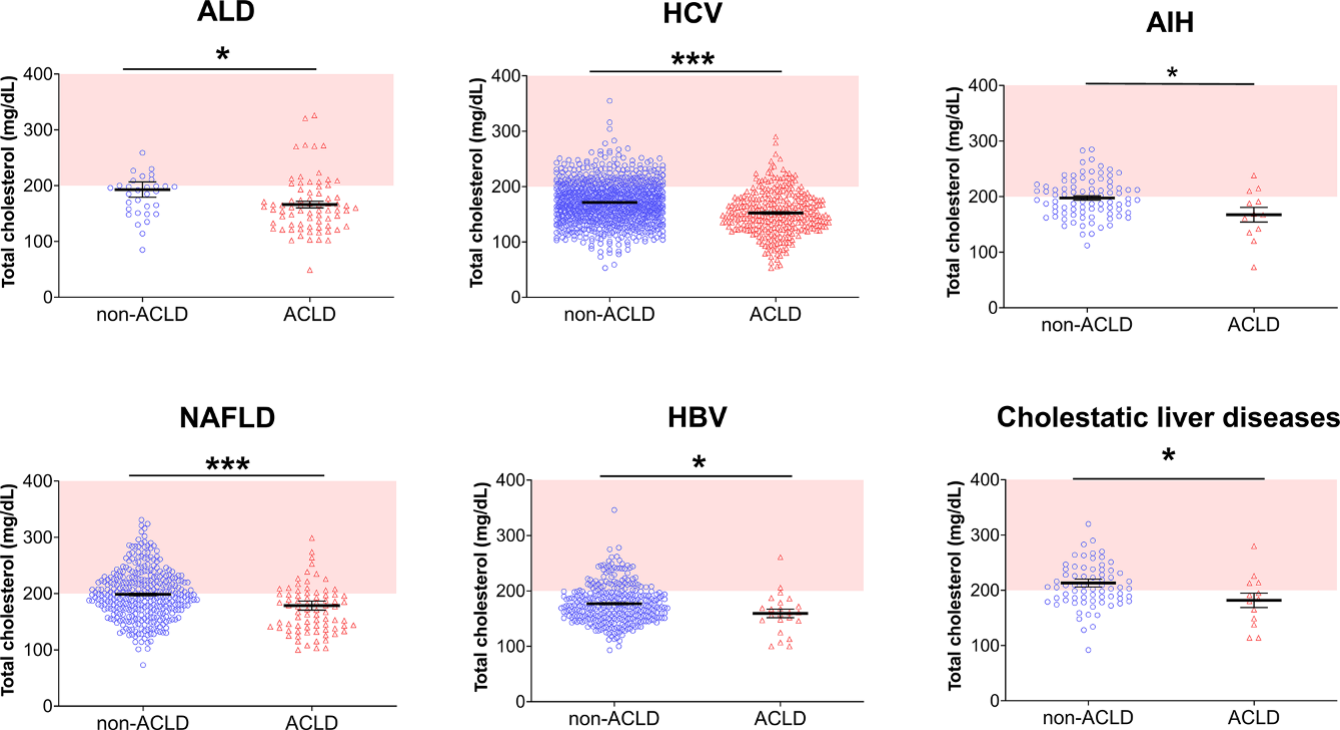
Scatter plots depicting individual total cholesterol levels for the respective etiology in non-ACLD (*blue*, *left*) and ACLD (*red*, *right*) patients. *p < 0.05, **p < 0.01, ***p < 0.001. *AIH* autoimmune hepatitis, *ALD* alcoholic liver disease, *CAP* controlled attenuation parameters, *HBV* hepatitis B virus infection, *HCV* hepatitis C virus infection, *NAFLD* non-alcoholic fatty liver disease

For analysis of systemic lipid levels, patients on lipid lowering therapy were excluded from the analysis. Fig. 2 shows lipid levels in the respective etiologies in non-ACLD and ACLD patients. Scatter plots showing correlation of total cholesterol levels with liver stiffness values of individual patients are provided as Supp. Fig. 1, both for the overall cohort as well as for subgroups of patients with different etiologies of liver disease.

When lipid status was analyzed according to etiology, total cholesterol was significantly lower in patients with ACLD compared to non-ACLD patients in ALD (191 (IQR 54) mg/dL vs. 160 (IQR 62) mg/dL; *p* = 0.015), HCV (170 (IQR 47) mg/dL vs. 149 (IQR 52) mg/dL; *p* < 0.001), HBV (172 (IQR 45) mg/dL vs. 161 (IQR 33) mg/dL; *p* = 0.031), NAFLD (197 (IQR 54) mg/dL vs. 172 (IQR 50) mg/dL; *p* < 0.001), cholestatic liver disease (205 (IQR 56) md/dL vs. 184 (IQR 70) mg/dL; *p* = 0.049) and in AIH (191 (IQR 47) mg/dL vs. 160 (IQR 69) mg/dL; *p* = 0.037), respectively; however, LDL values did not significantly differ in ALD (102 (IQR 44) mg/dL vs. 83 (IQR 41) mg/dL; *p* = 0.059) and cholestatic liver diseases (118 (IQR 50) mg/dL vs. 111 (IQR 49) mg/dL; *p* = 0.341) while LDL was significantly lower in ACLD patients with viral hepatitis (HCV: 93 (IQR 41) mg/dL vs. 79 (IQR 46) mg/dL; *p* < 0.001; HBV: 106 (IQR 59) mg/dL vs. 81 (IQR 50) mg/dL; *p* = 0.012), NAFLD (115 (IQR 43) mg/dL vs. 94 (IQR 54) mg/dL; *p* = 0.001) and AIH (125 (IQR 46) mg/dL vs. 85 (IQR 41) mg/dL; *p* = 0.034).

The HDL levels were significantly lower in HCV (50 (IQR 25) mg/dL vs. 44 (IQR 25) mg/dL; *p* < 0.001) and NAFLD-associated ACLD (51 (IQR 23) mg/dL vs. 42 (IQR 17) mg/dL; *p* < 0.001) in ACLD while it was not decreased in the other etiologies (ALD: 58 (IQR 35) mg/dL vs. 51 (IQR 24) mg/dL; *p* = 0.295; HBV: 46 (IQR 23) mg/dL vs. 42 (IQR 20) mg/dL; *p* = 0.271; cholestatic: 54 (IQR 35) mg/dL vs. 53 (IQR 51) mg/dL; *p* = 0.276; AIH: 58 (IQR 16) mg/dL vs. 40 (IQR 55) mg/dL; *p* = 0.524).

The presence of ACLD had no effect on triglyceride levels (ALD: 99 (IQR 74) mg/dL vs. 88 (IQR 53) mg/dL; *p* = 0.116; HCV: 97 (IQR 59) mg/dL vs. 90 (IQR 52) mg/dL; *p* = 0.077; HBV: 82 (IQR 66) mg/dl vs. 107 (IQR 97) mg/dL; *p* = 0.093; NAFLD: 119 (IQR 81) mg/dL vs. 108 (IQR 96) mg/dL; *p* = 0.626; cholestatic: 86 (IQR 56) mg/dL vs. 99 (IQR 50) mg/dL; *p* = 0.841; AIH: 100 (IQR 56) mg/dL vs. 86 (IQR 42) mg/dL; *p* = 0.417, respectively).

### Prevalence of hypercholesterolemia, increased LDL levels and hypertriglyceridemia in patients without lipid-lowering therapy

**Fig. 3:**
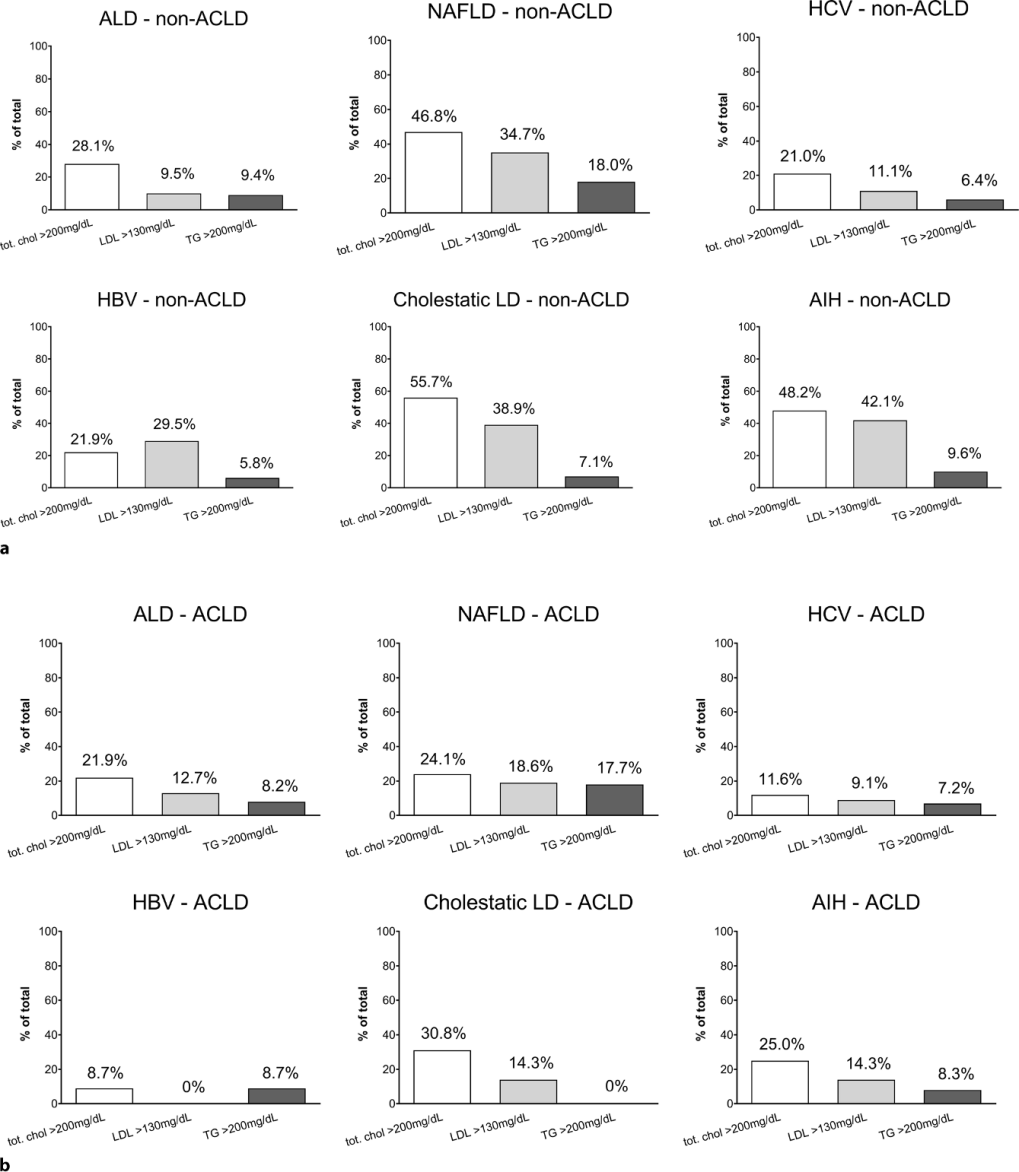
Percentages of patients with an increased total cholesterol level (*white bar*), increased LDL levels (*light grey*) or increased fasting triglyceride levels (*dark grey*), as indicated by the 2017 American Association of Clinical Endocrinologists and American College of Endocrinology guidelines. **a** non-ACLD patients; **b** ACLD patients. *AIH* autoimmune hepatitis, *ALD* alcoholic liver disease, *CAP* controlled attenuation parameters, *HBV* hepatitis B virus infection, *HCV* hepatitis C virus infection, *NAFLD* non-alcoholic fatty liver disease

As cut-off-values for increased CVD risk are well-defined in the AACE 2017 guidelines [11], we investigated the prevalence of hypercholesterolemia (total cholesterol >200 mg/dL), increased LDL levels (LDL >130 mg/dL) and hypertriglyceridemia (>200 mg/dL). There were important differences in the prevalence of hypercholesterolemia, increased LDL levels and hypertriglyceridemia between etiologies (Fig. 3). Interestingly, etiology-specific patterns of dyslipidemia remained similar between patient with and without ACLD. Cholestatic liver disease, AIH and NAFLD had the highest prevalence of dyslipidemia while patients with viral hepatitis and ALD showed the lowest prevalence of dyslipidemia.

## Discussion

Hyperlipidemia promotes atherosclerosis via endothelial cell activation and dysfunction. Due to ingestion of LDL by foam cells, plaques develop and subsequently lead to atherosclerotic lesions [12]. This leads to further secretion of inflammatory cytokines and accelerates plaque development [13]. While the mechanisms involved in plaque formation are relatively well-studied and the beneficial effects of pharmacologic intervention aiming at decreasing LDL levels in patients at risk for CVD/CVD patients are well-established, less evidence is available for the impact of dyslipidemia in patients with CLD and especially for the respective different etiologies of liver disease.

Certain etiologies, e.g. hepatitis C, may directly impact on CVD risk. The hepatitis C virus particles require LDL (and VLDL) receptors to enter hepatocytes [14] and cure of hepatitis C virus infection is associated with altered total cholesterol and LDL levels [15]. On the other hand, HBV does not seem to alter CVD risk [16]; however, the data on prevalence and severity of dyslipidemia are insufficiently comparable to other liver disease etiologies and differences in liver disease severity does not allow a fair comparison across different studies and patient cohorts. The data therefore add important insights as all patients were analyzed according to liver disease etiology and liver disease severity by reliable LSM. Nevertheless, patient numbers are low in some subgroups of patients with rare etiologies of liver disease, such as cholestatic or autoimmune liver disease. Although the numbers reflect the prevalence of these diseases, greater numbers would be favorable.

Although the risk for CVD in alcoholic liver disease seems to considerably depend on the actual amount of ingested alcohol, dyslipidemia has not been reported to represent an additional risk factor for CVD in ALD [17]. In cholestatic liver disease, where LDL cholesterol concentrations are usually elevated, CVD risk is not proportionally increased. These findings are explained by elevated lipoprotein X levels, which cannot be distinguished from LDL by standard laboratory tests but does not exert the same risk for atherosclerosis and thus, CVD events [18, 19]. These differences are of immense importance, as they implicate a more differentiated approach in treatment strategies. Even within the etiology of viral hepatitis, HCV and HBV have different risk profiles. The HCV-RNA circulates in large spherical particles, which bind in a competitive way with LDL and VLDL [20] and inhibition of microsomal triglyceride transfer protein by HCV infection leads to reduced VLDL secretion by hepatocytes, ultimately leading to decreased serum concentrations of VLDL and LDL [21]. In HBV, on the other hand, serum lipid levels are not affected [22], leading to higher absolute serum cholesterol levels than in HCV. Based on the findings in this large patient cohort, further research on the association between dyslipidemia, steatosis and CLD of different etiologies is warranted. While recent studies focused on NAFLD and NASH, dyslipidemia and its impact on the course of liver disease and cardiovascular risk should not be neglected in other etiologies of CLD. While the underlying mechanisms and associations between dyslipidemia, hepatic steatosis and CVD risk are being explored, a recent study by Corey and Chalasani showed that aggressive lipid lowering therapy reduces the high risk of CVD in NAFLD [23]; however, no specific recommendations are given for concomitant dyslipidemia and/or steatosis in other etiologies [23]. Importantly, the change of pathological lifestyle remains a management priority in patients with metabolic diseases [24].

In this study, patients with ACLD showed a relatively high prevalence of arterial hypertension and diabetes of >50% and 15%, respectively. Notably, however, patients with ACLD were on average older than non-ACLD patients, which may represent a potential confounder. These differences in baseline characteristics highlight the complexity of CVD risk in CLD. Future studies evaluating statin therapy in the CLD setting should therefore focus on CVD risk factors and take into account that CVD accounts for significant morbidity in CLD. Clinical guidelines should take these interactions into account and adapted cut-off values for recommendations to initiate lipid-lowering therapy might be necessary.

While we demonstrated differences in baseline characteristics between different etiologies including distinct patterns of dyslipidemia, we could demonstrate that the dyslipidemia patterns remain to be present when CLD progresses to ACLD. Nevertheless, it was observed that absolute cholesterol levels are lower in all etiologies after ACLD develops, reflecting impaired liver synthetic function. In this study, hepatic steatosis as assessed by CAP, increased in patients with ACLD, which is paralleled by an increase in metabolic comorbidities. Notably, few patients with any CLD other than NAFLD had a CAP ≥284 dB/m, implicating significant steatosis [25]. In theory, portal hypertension with increased bile acid levels and subsequent increased Takeda G‑protein-coupled receptor 5 (TGR5) activation might result in reduced steatosis in cirrhosis [26]. These findings highlight the fragile metabolic condition of NAFLD patients. Interestingly, also patients with HBV and cholestatic liver disease, who are thought to have no increased CVD risk compared to the general population [16, 27], still had a high prevalence of concomitant metabolic diseases that in turn may affect CVD risk profile. In summary, available studies that pool together different stages of CLD might underestimate the effect of stage-specific changes as well as etiology-specific risk modifications.

## Conclusion

Dyslipidemia is common in patients with CLD and significantly varies between different etiologies of liver disease. Progression of liver disease significantly alters lipid profiles, although etiology-specific patterns remain. It is warranted to systematically assess cardiovascular events after ACLD-induced “improvement” of dyslipidemia. Future studies should evaluate the impact of dyslipidemia and respective treatment on long-term outcome specifically in patients with CLD.

## Caption Electronic Supplementary Material

The electronic supplementary material contains a scatter plot with vizualized linear regression for total cholesterol and liver stiffness.

## Abbreviations

ACLD: Advanced chronic liver disease
AIH: Autoimmune hepatitis
ALD: Alcoholic liver disease
ALP: Alkaline phosphatase
ALT: Alanine aminotransferase
ASCVD: Atherosclerotic cardiovascular disease
AST: Aspartate aminotransferase
BMI: Body mass index
CAP: Controlled attenuation parameters
CLD: Chronic liver disease
CVD: Cardiovascular disease
GGT: Gamma-glutamyl transferase
HBV: Hepatitis B virus
HDL: High-density lipoprotein
HCV: Hepatitis C virus
IQR: interquartil range
LDL: Low-density lipoprotein
LSM: Liver stiffness measurement
NAFLD: Non-alcoholic fatty liver disease
TE: Transient elastography
