# Supplementary material for: Patterns and prevalence of dyslipidemia in patients with different etiologies of chronic liver disease
Source: Wien Klin Wochenschr. 2019 Sep 6;131(17):395–403. doi: 10.1007/s00508-019-01544-5 (PMC6748890; doi:10.1007/s00508-019-01544-5)
Supplement: Supplementary file 1 — The electronic supplementary material contains a scatter plot with vizualized linear regression for total cholesterol and liver stiffness. [file 508_2019_1544_MOESM1_ESM.docx]

**Supplementary information**

**Patterns and prevalence of dyslipidemia in patients with different etiologies of chronic liver disease**

by Unger Lukas W., Forstner Bernadette, Schneglberger Stephan, Muckenhuber Moritz, Eigenbauer Ernst, Scheiner Bernhard, Mandorfer Mattias, Trauner Michael, Reiberger Thomas.

**Supplementary Figure 1.**

**Scatter plot with linear regression (red line)**. Dots represent individual patients. Severity of liver fibrosis is indirectly correlated with total cholesterol values.
